# BPOZ-2 Gene Delivery Ameliorates Alpha-Synucleinopathy in A53T Transgenic Mouse Model of Parkinson’s Disease

**DOI:** 10.1038/srep22067

**Published:** 2016-02-26

**Authors:** Avik Roy, Suresh Babu Rangasamy, Madhuchhanda Kundu, Kalipada Pahan

**Affiliations:** 1 Department of Neurological Sciences, Rush University Medical Center, Chicago, IL, USA; 2 Division of Research and Development, Jesse Brown Veterans Affairs Medical Center, 820 South Darnen Avenue, Chicago, IL, USA

## Abstract

Ankyrin-rich BTB/POZ domain containing protein-2 or BPOZ-2, a scaffold protein, has been recently shown to control the degradation of many biological proteins ranging from embryonic development to tumor progression. However, its role in the process of neuronal diseases has not been properly explored. Since, abnormal clearance of metabolic proteins contributes to the development of alpha-synuclein (α-syn) pathologies in Parkinson’s disease (PD), we are interested to explore if BPOZ-2 participates in the amelioration of α-syn *in vivo* in basal ganglia. Here we report that lentiviral administration of *bpoz-2* gene indeed lowers the burden of α-syn in DA neurons in the nigra of A53T transgenic (A53T-*Tg*) mouse. Our detailed immunological analyses have shown that the overexpression of bpoz-2 dramatically improves both somatic and neuritic α-syn pathologies in the nigral DA neurons. Similarly, the specific ablation of *bpoz-2* by lentiviral-shRNA stimulates the load of monomeric and polymeric forms of α-syn in the nigral DA neurons of A53T-*Tg*. While investigating the mechanism, we observed that BPOZ-2 was involved in a protein-protein association with PINK1 and therefore could stimulate PINK1-dependent autophagic clearance of α-syn. Our results have demonstrated that bpoz-2 gene delivery could have prospect in the amelioration of alpha-synucleinopathy in PD and other Lewy body diseases.

Since, accumulation of α-syn is regulated by numerous signaling events involving many oxidative^1^, inflammatory^2^, metabolic^3^, epigenetic^4^ and neurotoxic factors^5^,^6^,^7^,^8^,^9^; identification of a specific molecular target is a major challenge in order to understand the molecular mechanism of alpha-synucleinopathy and develop an efficient therapy for the treatment of PD. BPOZ-2, an ankyrin repeat and BTB/POZ domain containing protein-2, serves as an important upstream regulator of wide-range of cellular pathways ranging from cell growth^10^ and development to apoptotic cell death^11^ and the clearance of metabolically defective proteins^12^. However, its role has never been explored in the amelioration of alpha-synucleinopathy *in vivo* in the nigra. It primarily serves as a growth suppressor protein, and because of its large size, it mainly participates in protein–protein interaction with wide range of intracellular proteins including ubiquitin ligase Cul-3 and ubiquitin. Its role as a scaffold protein mainly stems from the presence of ankyrin repeats (ARs) and two BTB/POZ domains that render BPOZ to function as an interface between two proteins^12^.

Amelioration of α-syn is a complex cellular event that involves both ubiquitin-proteasome and autophagic pathways^13^. Recent evidence suggests that the generation of alpha-synucleinopathy in DA neurons is directly involved in the impairment of cellular autophagy^14^. Therefore autophagic impairment is often considered as one of the primary contributing factors of α-syn aggregation^15^. PINK1 has been reported to play a crucial role in the autophagic clearance of damaged mitochondria^16^. Evidently, mutation and loss of function of pink1 gene has been shown to be involved in PD^17^. However, overexpression of pink1 gene failed to reproduce the regeneration of DA neurons^18^ suggesting the demand of more precise molecular target that modulates the activity of PINK1.

Earlier we have shown that BTB/POZ domain containing protein-2 could be responsible for lowering the expression of α-syn in the cultured DA neurons^19^. However, the difficulties in the establishment of α-syn pathologies in cultured DA neurons as well as its lack of pathological relevance with actual disease restrict the scope of our previous report. Therefore, here we wanted to validate the effect of bpoz-2 gene manipulation in the amelioration of alpha-synucleinopathy *in vivo* in the nigra of adult A53T-tg mouse brain. Here we report that lentiviral administration of bpoz-2 gene indeed lowers the burden of α-syn in DA neurons in the nigra of A53T-Tg mouse. Our detailed immunohistochemical and immunoblot analyses have clearly shown that the overexpression of bpoz-2 dramatically improves both the somatic and neuritic α-syn pathologies in the nigral DA neurons. Similarly, the specific ablation of bpoz-2 by lentiviral-shRNA fails to ameliorate, but strongly increases the load of monomeric and oligomeric forms of α-syn in the nigral DA neurons of A53T-Tg mice as revealed by different immunological analyses. While investigating the mechanism, we observed that BPOZ-2 could be involved in PINK1-dependent autophagic clearance of α-syn. Our results have demonstrated that bpoz-2 gene delivery could be prospective in the amelioration of alpha-synucleinopathy in PD and other Lewy body diseases.

## Results

### Expression of BPOZ-2 is strongly down-regulated in the nigra of A53T-Tg mouse

**Figure 1:**
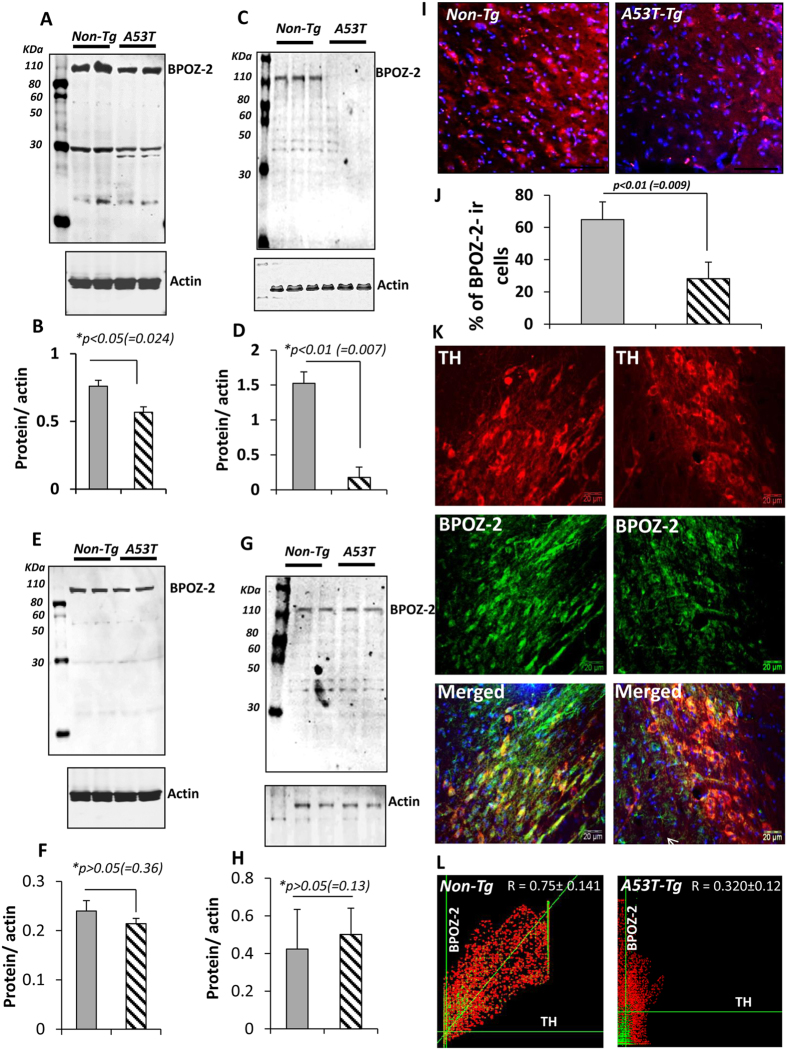
Analysis of BPOZ-2 expression in the nigra and striata of A53T-Tg mouse brain. (**A**) Immunoblot analyses of BPOZ-2 protein were performed in the nigral extracts of 3 months old A53T-Tg and age-matched Non-Tg mice as shown in duplicates (top) and then normalized with actin (bottom). According to the product sheet of BPOZ- antibody (Cat# ab112610; Abcam), the predicted band comes around 100 KDa with a minor band around 30 KDa. (**B**) Analyses were further confirmed by relative densitometric analyses with image J software. *p < 0.05 vs. nigral expression of bpoz-2 in Non-Tg mice (**C**) The expression of BPOZ-2 was analyzed in the nigral extracts of 8 months old male A53T-*Tg* and Non-Tg mice (*top*) with actin (*bottom*). (**D**) Results were further confirmed with densitometric analyses (*n* = *3*). *p < 0.01 vs. BPOZ-2 of Non-Tg. Similarly BPOZ-2 expression was analyzed in the striatal homogenates of (**E**) A53T-Tg and (**G**) Non-Tg mouse brain followed by densitometric quantification of blots (**F**,**H**). (**I**) Immunohistochemical analyses of BPOZ-2 in the nigra of 8 months old Non-Tg and A53T-tg mouse brains. (**J**) Quantification of BPOZ-2-immunoreactive (ir) cells followed by normalization with DAPI-positive cells of respective image (n = 10 per group). (**K**) Dual immunohistochemical analyses of BPOZ2 (green) with tyrosine hydroxylase (TH; red) in the nigral sections of 3 months old Non-tg (left) and A53T-tg (right) mouse brain. (**L**) Co-localization of TH and BPOZ-2 was further analyzed by colocz tool of NIH-FIJI software. Mean Pearson coefficient was displayed on the figure after analyses of 10 different images of five animals per group.

Previously we had shown that the down-regulation of BPOZ-2 could be involved in the accumulation of α-syn in DA neurons^19^. However, the expression of BPOZ-2 has never been tested *in vivo* in the nigra of A53T-*Tg* mouse that exhibits a spontaneous and aggressive accumulation of human α-syn as early as three months of age and continues to increase in an age-dependent manner. Therefore, we wanted to study the expression of BPOZ-2 *in vivo* in the striata and nigra of both 3 months and 9 months old A53T-*Tg* mouse. The expression of BPOZ-2 protein was analyzed by immunoblot analyses from nigral or striatal tissue homogenates and then compared with age-matched non-transgenic (Non-*Tg*) wild type animals. Interestingly, the expression of BPOZ-2 protein was significantly lower (*p < *0.05;* = *0.024*) in the nigra of 3-months old A53T-*Tg* transgenic mice (Fig. 1A,B). The nigral expression of BPOZ-2 was continued to decrease with age and found to be strongly reduced (*p < *0.01*; *=* *0.007*) in 9-months’ old male A53T-Tg mice (*n* *=* *3*) compared to wild type mice (Fig. 1C,D) suggesting that A53T-Tg is associated with strong age-dependent reduction of BPOZ-2 in their nigra. However, its expression remained unchanged in the striata when compared between A53T-*Tg* and Non-*Tg* animals of 3 months (Fig. 1E,F) and 9 months (Fig. 1G,H) of age as evident by immunoblot and subsequent densitometric analyses. Consistently, our immunohistochemical analyses (Fig. 1I) followed by our counting studies (Fig. 1J) clearly revealed that the expression of BPOZ-2 was indeed very low in the nigra of A53T-*Tg* mice compared to Non-*Tg* mice. Our dual immunostaining analyses of tyrosine hydroxylase (TH; red) and BPOZ-2 (green) (Fig. 1K) followed by co-localization analyses in Fiji colocz tool (Fig. 1L; right) further indicated that nigral DA neurons of A53T-*Tg* animals expressed very low amount of BPOZ-2 with a low, but positive Pearson coefficient (*0.320* ± *0.122 from 10 different images*) (Fig. 1K; right), whereas neuronal expression of BPOZ-2 remained high in the nigra of Non-*Tg* mice (Fig. 1K; left), which was reflected by a high Pearson coefficient (*0.75* *±* *0.141 from 10 different images*) (Fig. 1L; left). Therefore, collectively our results suggest that the expression of BPOZ-2 is mainly observed in the nigrostriatum and that expression is strongly downregulated in the nigra, but not in striata, of A53T-Tg mouse with increasing age.

### Role of BPOZ-2 in the regulation of alpha-synucleinopathy in the nigra of A53T-Tg mice

**Figure 2:**
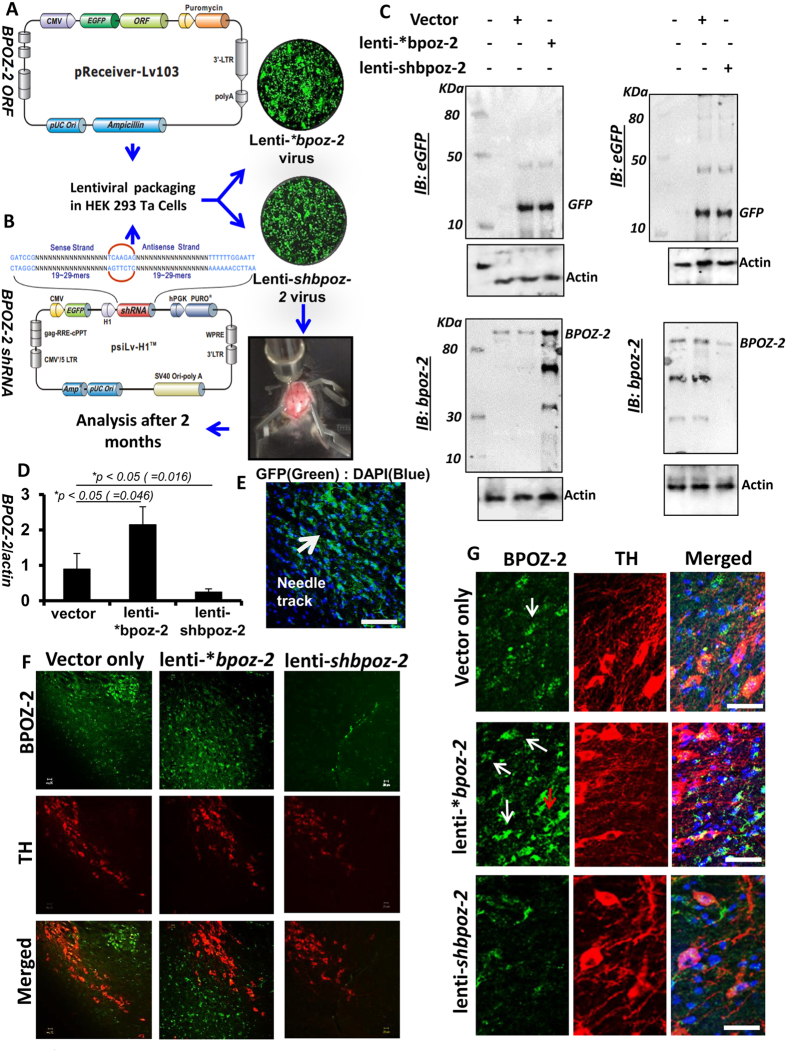
Lentiviral administration of bpoz-2 ORF and shRNA in the nigra of A53T-tg mouse brains. (**A,B**) A schematic diagram showing Lentiviral plasmid maps for GFP-linked BPOZ-2 ORF (top) and shRNA (bottom),a short description for packaging these plasmids in virus particles with the help of HEK293Ta packaging cells, and gene delivery strategy in mouse brain. Images were shown as HEK293Ta cells transduced with BPOZ-2 ORF (top) and shRNA (bottom) to analyze their transduction efficiency. After that 10^6^ viral particles were injected in the nigra of 8 months old A53T-Tg mouse brain (n = 10 per group) followed by analyses of our results after another two months (**C**) Immunoblot analyses of GFP (top) and BPOZ-2 (bottom) in empty vector- (lenti-vector), bpoz-2 ORF-(lenti-*bpoz-2), and bpoz-2 shRNA (lenti-shbpoz-2)-infused nigral extracts of A53T-Tg mouse brains after one month of injection. (**D**) densitometric analyses were performed combining all three groups under the same plot.(**E**) Needle track and (**F**) establishment of bpoz-2 genes were shown by dual immunostaining with TH (red) and BPOZ-2 (green). (**G**) Magnified view of these images to display the distribution of BPOZ-2 (green) in nigral sections stained with TH (red) in different groups of animals. Results were confirmed after analyzing three different experiments.

**Figure 3:**
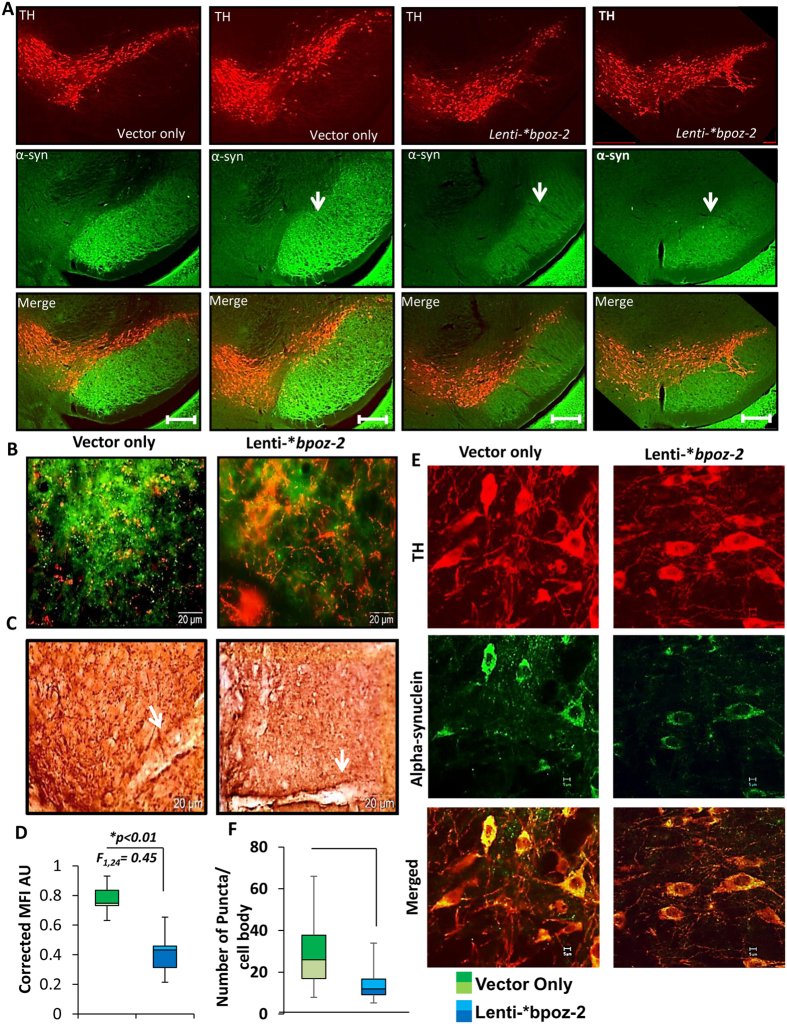
Effect of Lentiviral administration of bpoz-2 ORF on the expression of α-syn in the nigra of A53T-Tg mouse. (**A**) Dual immunostaining of TH (red) and α-syn (green) in the nigra of lenti-vector (first two columns) and lenti-*bpoz2 (last two columns) –infused brains of A53T-Tganimals. Pictures were taken in 4× objective lenses of fluorescence microscope with detailed distribution of α-syn in SNpr region. Scale bar 50 μm. (**B**) 20× Magnified view of SNpr regions of lenti-vector and lenti-*bpoz-2 nigral sections. (**C**) DAB staining of SNpr regions of lenti-vector and lenti-*bpoz-2 animals. (**D**) Mean Fluorescence Intensity of neurites at SNpr regions of lenti-vector and lenti-*bpoz-2 animals were calculated, adjusted with non-nigral α-syn intensities for background correction and compared in a box plot analysis. Results are mean ± SEM of 13 different images per group taken from five different animals. (**E**) Somatic expression of α-syn was monitored by dual-immunolabeling of TH (red) and α-syn (green) in the SNpc regions of nigra in Lenti-vector and lenti-*bpoz-2 groups of animals. (**F**) Box plot analyses of somatic α-syn-ir (immunoreactive) puncta in 13 different images of five different animals of lenti-vector and lenti-*bpoz-2 group. *p < 0.01 vs. lenti-vector group. White arrow indicates the injection site.

Since the accumulation of α-syn increases with age, next we wanted to explore if BPOZ-2 was involved in the nigral accumulation of α-syn. In order to investigate the direct and physiological role of BPOZ-2 in the regulation of α-syn expression in nigra, we adopted a lentivirus-mediated gene delivery strategy by which we either overexpressed or knocked-down *bpoz-2* gene in the nigra of 7–8 months’ old male A53T mice followed by a series of immunological analyses to measure the nigral load of α-syn. We first cloned and then packaged GFP-linked empty vector (*Lenti-vector*), *bpoz-2* ORF cDNA (Lenti-*bpoz-2) (Fig. 2A), and *bpoz-2* shRNA *(Lenti-shbpoz-2)* (Fig. 2B) in lentivirus by infecting HEK293 Ta cells as mentioned elsewhere^20^. After that, these engineered viral particles were injected (Fig. 2) in the nigra of 7–8 months old male A53T-Tg animals (*n* *=* *10 per group*), maintained for two more months to establish the genes, and then the analyses of bpoz-2 gene expression in nigra to validate our gene delivery efficiency. Accordingly, we observed that viral particles engineered with empty vector, bpoz-2 ORF cDNA (Fig. 2C; left), and bpoz-2 shRNA (Fig. 2C; right) efficiently delivered and established manipulated genes in the nigral tissue as evident by GFP (Fig. 2C; top) and BPOZ-2 (Fig. 2C; bottom) immunoblots as we observed that ORF-cDNA upregulated and shRNA inhibited the expression of BPOZ-2. Consistently, our combined densitometric analyses (Fig. 2D) dual immunohistochemical analyses (Fig. 2E,G) revealed that after lentiviral delivery, the expression of BPOZ-2 was strongly upregulated in TH-*ir* DA neurons of nigra in lenti-*bpoz-2 group, whereas the expression was significantly knocked-out in lenti-shBPOZ-2 group of A53T-Tg animals. Next, we wanted to study if the lentiviral overexpression of bpoz-2 inhibited the burden of α-syn in nigra. Interestingly, our dual immunostaining analyses of TH (red) and α-syn (green) indicated that the overexpression of BPOZ-2, but not the administration of vector alone, significantly attenuated the load of α-syn in the neurites at substantia nigra pars reticulate (SNpr) region (Fig. 3A,B). Moreover, the α-syn signal intensity in extra-nigral region remained same in all cases as indicated in the bottom-right corner of the FITC (green) panel. Since photo-bleaching of florescence signal could impact our conclusion, we performed DAB staining of α-syn in the nigral sections of *lenti-vector* and lenti-*bpoz-2 groups of animals (*n* = *5 per group*). Consistently, we observed that the administration of lenti-*bpoz-2, but not *lenti-vector*, strongly attenuated the burden of α-syn in the fibers of SNpr regions (Fig. 3C). Next, we measured neuritic densities of α-syn in both *lenti-vector* and lenti-*bpoz-2 groups of animals (*n* = *5 per group*) neurites of SNpr. Neuritic density was measured and plotted (Fig. 3D) after calculating the mean fluorescence intensities (MFI) of 13 different images per group (data collected from five animals; *n* = *5*) followed by the correction with the MFI of non-nigral (here hippocampal) α-syn. Our analyses clearly indicated that overexpression of bpoz-2 significantly reduced (*p* < *0.01; F*_*1, 24*_ = *45*) the neuritic burden of α-syn compared to control group. However, somatic burden of α-syn contributed significantly to the neurodegenerative pathology of DA neurons as well. Our dual immunostaining analyses of TH (red) and α-syn (green) indicated that the administration of lenti-*bpoz-2, but not lenti vector, significantly attenuated the level of α-syn in DA cell bodies of nigra (Fig. 3E). To further confirm our result, we performed quantitative analyses of α-syn inclusions in cell bodies of DA neurons by counting number of puncta enclosed in a cell body. Briefly, each individual cell was opened, magnified in Olympus microsuite software, and then counting analysis was executed with the help of touch counting module. Consistently, lenti-*bpoz-2, but not *lenti-vector* animals, showed significant improvement in lowering the somatic burden of α-syn puncta in nigral DA neurons (Fig. 3F) indicating the beneficial role of *bpoz-2* in ameliorating the load of α-syn in DA neurons.

**Figure 4:**
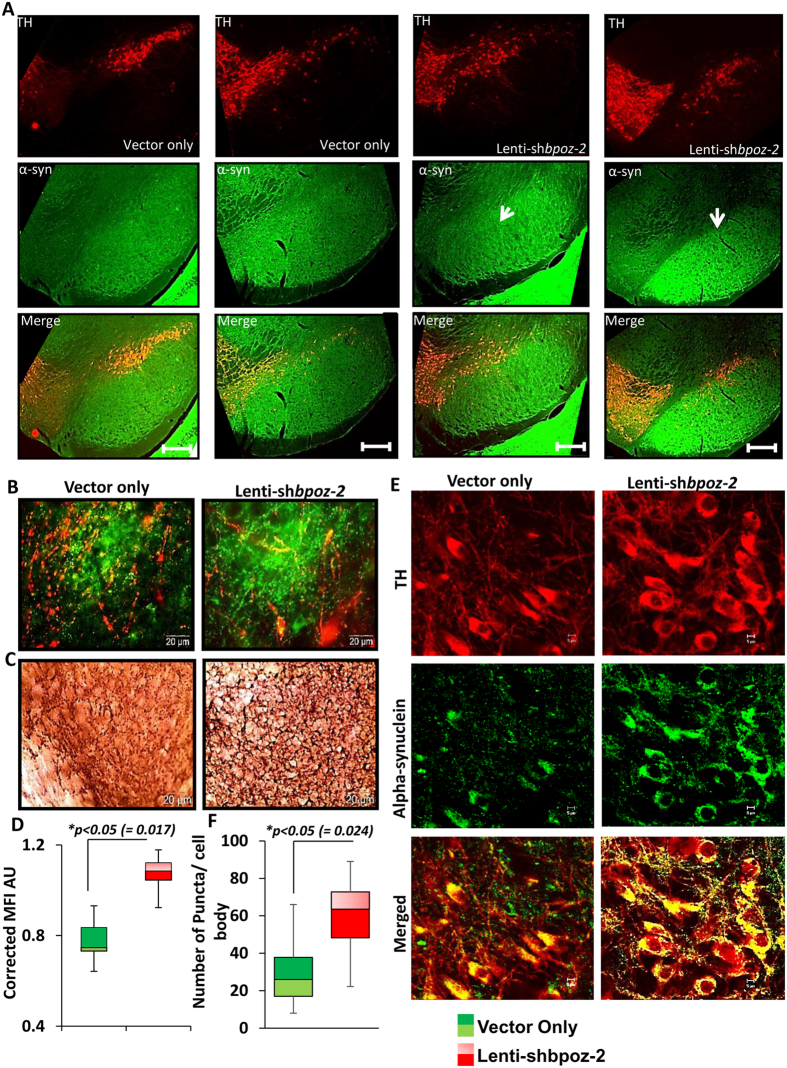
Effect of Lentiviral administration of bpoz-2 shRNA on the expression of α-syn in the nigra of A53T-Tg mouse. (**A**) Dual immunostaining of TH (red) and α-syn (green) in the nigra of lenti-vector (first two columns) and lenti-shbpoz2 (last two columns) –infused brains of A53T-Tganimals. Pictures were taken in 4× objective lenses of fluorescence microscope with detailed distribution of α-syn in SNpr region. Scale bar 50 μm. (**B**) 20× Magnified view of SNpr regions of lenti-vector and lenti-shbpoz-2 nigral sections. (**C**) DAB staining of SNpr regions of lenti-vector and lenti-shbpoz-2 animals. (**D**) Mean Fluorescence Intensity of neurites at SNpr regions of lenti-vector and lenti-shbpoz-2 animals were calculated, adjusted with non-nigral α-syn intensities for background correction and compared in a box plot analysis. Results are mean ± SEM of 13 different images per group taken from five different animals. (**E**) Somatic expression of α-syn was monitored by dual-immunolabeling of TH (red) and α-syn (green) in the SNpc regions of nigra in Lenti-vector and lenti-shbpoz-2 groups of animals. (**F**) A quantitative Box plot analysis of somatic α-syn-ir (immunoreactive) puncta in 13 different images of five different animals of lenti-vector and lenti-shbpoz-2 group. *p < 0.05 vs. lenti-vector group. White arrow indicates the injection site.

**Figure 5:**
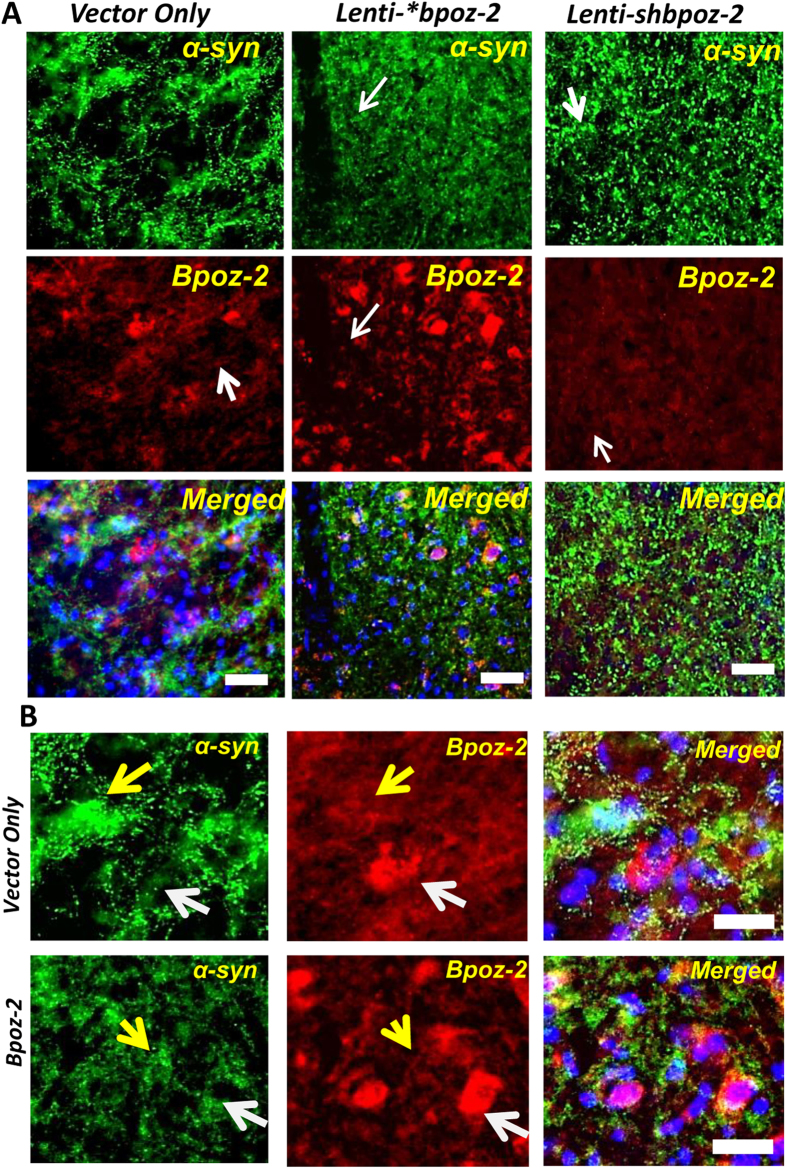
Analyzing the direct relationship between the expression of bpoz-2 and α-syn in the nigra of A53T-Tg animals. (**A**) Dual Immunohistochemical analyses of α-syn (green) and bpoz-2 (red) in the nigra of lenti-vector (left), lenti-*bpoz-2 (middle) and lenti-shbpoz-2 groups of animals. Images were taken under 20× objective of Olympus fluorescence microscope. (**B**) Magnified view of micrographs under 100× micrographs showing detailed intracellular distribution of α-syn and BPOZ-2.

Next, we knocked-down the endogenous expression of *bpoz-2* by delivering lentiviral shRNA of bpoz-2 (*lenti-shbpoz-2*) in the nigra of 6–8 months old male A53T-Tg animals (*n* = *10*). After two months of cerebral injection, animals were analyzed for the expression of α-syn. Our dual immunohistochemical studies clearly showed that the neuritic burden of α-syn in the *SNpr* region was significantly upregulated after bpoz-2 shRNA administration when compared with lenti-vector group (Fig. 4A). Since, lower magnification images were difficult to comprehend the effect of shRNA, SNpr region was further magnified under microscope, captured, and displayed to understand the effect of knocking-down of endogenous level of bpoz-2 (Fig. 4B). As evident from the magnified image, the administration of lenti-shbpoz-2, but not *lenti-vector*, upregulated the fibrillar load of α-syn puncta. We further corroborated our result by measuring the MFI of α-syn puncta from entire SNpr region followed by adjusting with MFI of hippocampal α-syn from respective images (total 13 images from five animals per group) and then presented in a box plot (Fig. 4D). Our box plot analyses confirmed that SNpr region of *lenti-shbpoz-2* animals was associated with significantly higher density of α-syn puncta compared to the *lenti-vector* group. Similarly, our DAB staining method further validated our finding as we observed that knocking-down of bpoz-2 strongly increased the density of fibrillar puncta in the SNpr region (Fig. 4C). Consistent to our previous finding, we observed that lenti-shbpoz-2 strongly upregulated the number of α-syn-ir somatic inclusions in the DA neuronal cell bodies of SNpc region as shown in fig 4E. Accordingly, our quantification analyses of somatic inclusions of α-syn followed by boxplot analyses (Fig. 4F) further confirmed our finding. Next, we performed a double immunostaining of BPOZ-2 (red) and α-syn (green) to establish the direct relationship between two proteins (Fig. 5A). Interestingly, the BPOZ-2–ir cell bodies in lenti-^*^bpoz-2 animals were observed to be associated with less burden of α-syn as indicated by white arrowhead (Fig. 5B), whereas the absence of BPOZ-2 –ir cells were observed with heavy burden of α-syn inclusions in the nigra of indicated by yellow arrowhead, suggesting that expression of BPOZ-2 is crucial for the amelioration of α-syn inclusions. Accordingly, nigral section of lenti-shbpoz-2 animals displayed heavy load of α-syn with no bpoz-2 reiterating that BPOZ-2 is directly involved in the amelioration of α-syn in nigra.

### Role of BPOZ-2 in the aggregation of α-syn in the nigra of A53T-Tg mice

**Figure 6:**
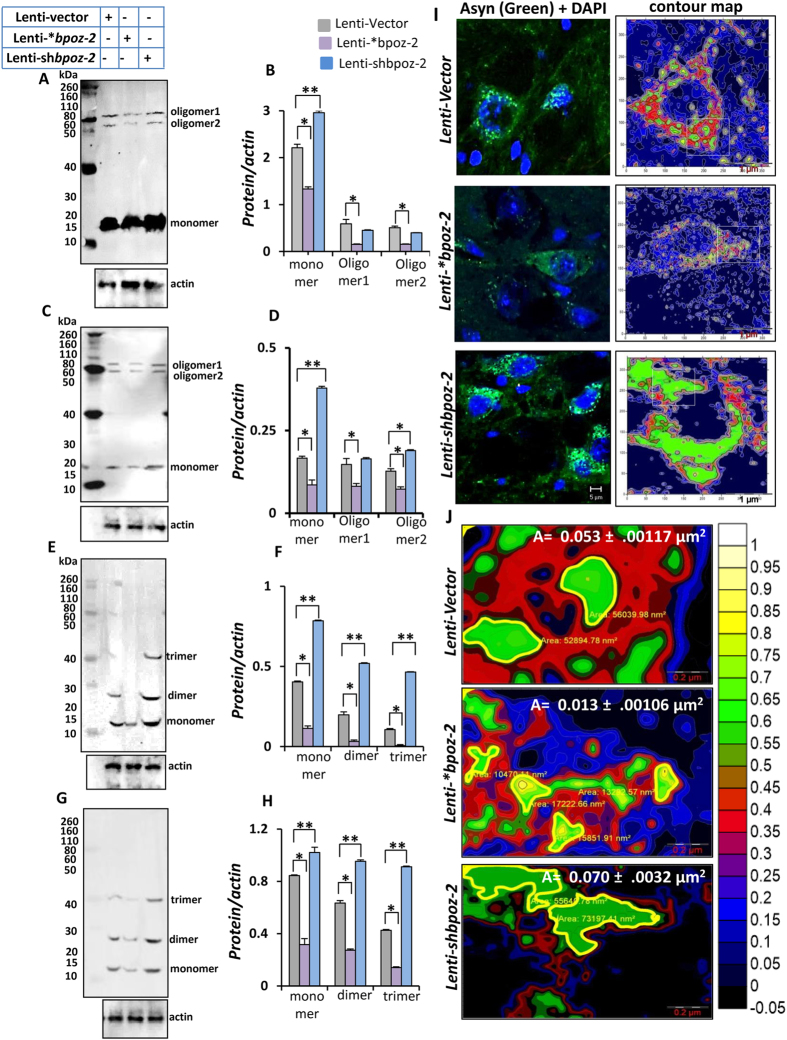
Effect of lentiviral constructs of bpoz-2 ORF and shRNA on the aggregation of α-syn in the nigra of A53T-Tg mouse. (**A**,**C**,**E**,**G**) Immunoblot analyses of α-syn in the nigral homogenates of lenti-vector, lenti-*bpoz-2 and lenti-shbpoz-2 animals. (**B**,**D**,**F**,**H**) Relative densitometric analyses of respective Immunoblots. *p < 0.05 vs. lenti-vector and **p < 0.01 vs. lenti-vector. Each immunoblot represents the result obtained from independent experiments of three groups of animals. First two immunoblots (**A**,**C**) were performed with Millipore α-syn antibodies and last two immunoblots (**E**,**G**) were performed with Abcam antibodies. (**I**) High-resolution images of nigral DA neurons in three different groups showing the polymeric puncta in the cell bodies (left column). Individual puncta was displayed in soma after contour modification of DA cell body (right column) in each group. (**J**) Area of α-syn-ir puncta was calculated and displayed on the top of each image as mean ± SEM of 10 different images. Calculation was done in Olympus Microsuite 5 software after adjusting the scale bar with original raw image.

So far, we analyzed the effect of *bpoz-2* on the regulation of α-syn expression. However, it is not clear if bpoz-2 is involved in the aggregation or polymerization of α-syn, which is a pathologically relevant to Lewy body-associated PD. The best method to analyze the polymerization of α-syn is immunoblot analysis. We performed our immunoblot analyses with two different α-syn antibodies. MJFR1 clone of α-syn antibody is best to analyze monomer and oligomeric α-syn, whereas AB5336P antibody of α-syn from Millipore detects polymeric form of α-syn. Interestingly, lentiviral overexpression of bpoz-2, but not empty vector, significantly attenuated the polymerization (Fig. 6A–D) and di- or trimerization (Fig. 6E–H) of α-syn, which we further confirmed by densitometric analyses. Another way to confirm the polymerization of α-syn is to measure area of α-syn-ir puncta. However, measuring area of individual puncta could be erroneous as the fluorescent signal generated from each puncta overlaps with each other, which generates a masking effect. To circumvent this problem, we magnified each cell 100 times under microscope and then reconstructed them with contour as shown in second column of fig 6I. Briefly, α-syn-*ir* cell bodies (Fig. 6I; first panel) were zoomed under microscope, opened in a contour module of Surfer software package (Golden Software, LLC; Colorado). The entire cell provided a series of contours with different size and intensities displayed in green color. Intensity of each contour could be evaluated based on the number of radial lines inside each green enclosures, whereas overall intensity of α-syn signal was shown in a color scale from -0.05 to 1. Quantifying each contour provides the number of inclusions, whereas measuring the perimeter of outermost radial line (Thick dark line around green enclosure: Fig. 6J) provided the area of each inclusion. Area of α-syn inclusion was calculated in the Microsuite 5 software after drawing a fitted polygon around each contour. Area of α-syn inclusions were counted from 25 images from each group and presented as area ± SEM on the top of the image. Therefore, based on our immunoblot and other quantitative analyses, we confirmed that the lentiviral overexpression of bpoz-2 not only downregulaed the expression, but strongly down-regulated the aggregation or polymerization of α-syn, whereas lentiviral knocking down of bpoz-2 stimulates both the expression and the aggregation of α-syn.

### BPOZ-2 requires PINK1 for the amelioration of α-syn in the nigra of A53T-Tg mice

**Figure 7:**
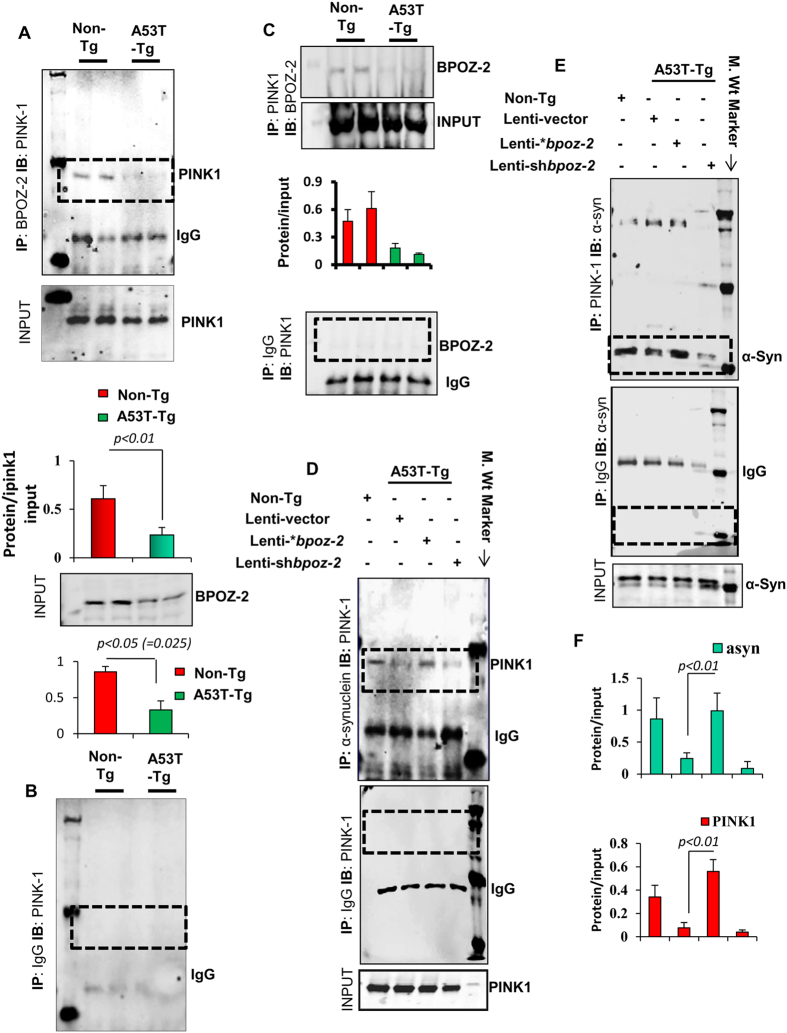
Evaluating the association between BPOZ-2 and PINK1 in the nigra of A53T-Tg mice. (**A**) Immunoprecipitation (IP) of BPOZ-2 followed by immunoblot (IB) analysis of PINK1 was performed in the nigral extracts of Non-Tg and A53T-Tg (n = 3/group) animals (*top*).Densitometric analyses were performed by normalizing with PINK1 and BPOZ-2 inputs in ImageJ software. (**B**) IP followed by IB analyses of PINK1 in IgG-pulled-out nigral extract served as negative control (*middle*). (**C**) IP of PINK1 followed by IB of BPOZ-2 was analyzed with densitometric analysis (middle) and IgG pulled-down fraction (bottom). (**D**) IP by α-syn followed by IB of PINK1 (*top*) in the nigral homogenates of non-tg-, lenti-vector-, lenti-*bpoz-2- and lenti-shbpoz-2-injected animals. IP with IgG and input were included as control. (**E**) IP of PINK1 followed by IB of α-syn in the nigral tissue of non-tg-, lenti vector-, lenti-*bpoz-2-, and lenti-shbpoz-2-injected animals was performed. IP with IgG and input were included. (**F**) Densitometric analyses of α-syn after pulling down with PINK1 (*top*) and similar analysis of PINK1 after pulling down with α-syn were performed (*bottom*). Results are mean ± SD of three different analyses.

Next, we wanted to study the mechanism by which BPOZ-2 ameliorated α-syn burden from nigral neurons. In our previous manuscript^19^, we predicted that BPOZ-2 could interact with ubiquitin ligase Parkin. However, our immunoprecipitation (IP) analyses indicated that there was no detectable interaction with Parkin (data not shown). Interestingly, our IP with BPOZ-2 followed by IB with PINK1study revealed that there was a strong interaction of BPOZ-2 and PINK1 in the nigra of Non-Tg animals (*n* = *3*), whereas that interaction was significantly compromised in the nigra of A53T-Tg nigra (*n* = *3*) (Fig. 7A; top panel), which we confirmed with densitometric analyses (Fig. 7A; bottom panel). We further validated our finding with IP against IgG (Fig. 7B). The endogenous level of BPOZ-2 is low in A53T-tg animals. Low interaction between BPOZ-2 and PINK1 could be due to low endogenous level of BPOZ-2. To nullify that concern, we have included the input of BPOZ-2 and then normalized PINK1 result with that input by performing relative densitometric analyses. We observed that detected level of PINK1 is still significantly low in A53T-tg compared to Non-tg animals suggesting that the association of PINK1 and BPOZ-2 does not depend on the endogenous deficit of BPOZ-2 in A53T-tg animals. Next, we wanted to study the interaction between BPOZ-2 and PINK1 by IP with PINK1 followed by IB with BPOZ-2. Consistently, we observed that BPOZ-2 and PINK1 interaction was severely compromised in A53T-tg nigra (Fig. 7C). Next, we analyzed the role of this interaction in the regulation of alpha-synucleinopathy in the nigral tissue of lenti-vector, lenti*-* *bpoz-2, and lenti-shbpoz-2 animals. Interestingly, our IP analysis indicated that the interaction of α-syn and PINK1 increased in the nigra of lenti-*bpoz-2, but not lenti-*vector* animals (Fig. 7D,F) suggesting that overexpression of bpoz-2 could be involved in enhanced interaction of αsyn with PINK1. On the other hand, the interaction between α-syn and PINK1 was significantly reduced in lenti-*shbpoz-2* animals when compared with lenti-vector group (Fig. 7E,F) suggesting that the absence of bpoz-2 impairs the interaction of α-syn with PINK1. Since PINK1 is involved in the clearance of damaged protein via selective autophagy, BPOZ-2 could be involved in the PINK1-mediated autophagic clearance of α-syn in A53T-tg animals.

### Effect of BPOZ-2 gene manipulation on the restoration of behavioral deficits of A53t-tg animals

While monitoring gross motor activity and other basal ganglia-controlled motor performances including rota-rod, stereotypy and open-field activity; unfortunately, we did not observe any significant differences between lenti-shbpoz-2 and lenti-*bpoz-2 group. Although overexpression of bpoz-2 slightly improved motor performances such as horizontal activity (Supplementary Figure 1A), total distance (Supplementary Figure 1B), stereotypy counts (Supplementary Figure 1C), and rotarod (Supplementary Figure 1D) in A53T-tg animals, but all these data failed to cross the significance level of p < 0.05. One possibility is animals included in this study were more than 8 to 9 months old with established disease pathology. Their gross motor activities had already been compromised severely. However this is a preliminary and qualitative finding that needed more extensive analyses with more number of animals and different age group. On contrary, we observed that BPOZ-2 overexpression restored the dopamine level significantly suggesting that the overexpression of bpoz-2 gene can recover the molecular deficits of DA neurons of A53T-tg animals (supplementary Figure1E,F). Kruz *et al.*^21^ reported that the old A53T-SNCA overexpressing mice exhibit a diminished DA response, despite cellular efforts to maximize DA signaling. Therefore, upregulated DA might not be sufficient to restore the motor deficits. Moreover, Mendritzki^22^ etal and others reported that old A53T-tg animals are associated with extensive spinal cord pathology that compromises the motor performance of older animal in a large extent. Viral manipulation of bpoz-2 gene only improves the central pathology, but not the peripheral deficits in this case. However, our observation was made after analyses in three animals. Therefore, more animals are needed to propose this observation statistically significant. Our future plan is to monitor if BPOZ-2 gene delivery is able to restore the health of DA neurons in relatively younger animals and can improve the longevity of these animals.

## Discussion

Accumulation of α-syn is an important pathological hallmark for both sporadic and familial PD. Therefore, the clearance of this molecule has been looked upon with high importance. However, the mechanism involved in the amelioration of α-syn is not properly understood. In our previous report, we have shown that BPOZ-2 could play an important role in the downregulation of α-syn in cultured DA neurons^19^. However, its role cannot be warranted unless tested *in vivo* in an animal model with progressive α-syn pathology. There are several reasons for validating our observation in an animal model. *First*, the accumulation of α-syn in PD brain is a progressive event, which worsens with increase in age. That phenomenon cannot be replicated in a regular cell-culture experiment. *Second*, Different factors including glial activation, inflammation and other metabolic abnormalities contributed by non-neuronal cells have been shown to be involved in the accumulation of α-syn in nigral DA neurons, which is not possible to demonstrate in pure cell culture study. In our present study, we have tested the role of BPOZ-2 in the amelioration of α-syn in 8–9 months old male A53T-tg mouse, which is generated in the laboratory of Dr.Virginia Lee^23^. Our different protein studies clearly revealed that the basal expression of BPOZ-2 was significantly less in the nigra of 8–9 months old A53T-tg animals, compared to the nigra of age-matched Non-Tg animals. Moreover, the nigral expression of BPOZ-2 had been found to be downregulated with increasing age of A53T-tg animals. Since, the accumulation of α-syn worsens with increasing age in these animals; we predicted that the age–dependent downregulation of BPOZ-2 could be involved in the accumulation of α-syn in A53T-tg animals. To test that hypothesis, we performed lentivirus-mediated gene delivery strategy of bpoz-2 gene and studied its effect in the regulation of α-syn in 8–9 months old male A53T-tg animals.

Several lines of our studies indicated that lentiviral overexpression of bpoz-2 attenuated the expression of α-syn in nigra. *First*, our dua-limmunostaining of TH and α-syn showed a significant downregulation of α-syn in the cell bodies of DA neurons in SNpc of lenti-*bpoz-2 A53T-tg animals. Second, similar analyses revealed the attenuation of neuritic burden of α-syn in the of SNpr region these animals. *Third*, our immunoblot analyses indicated that lentiviral administration of bpoz-2 gene downregulated the expression of monomeric, dimeric, trimeric and oligomeric forms of α-syn in A53T-tg animals. *Fourth*, different quantitative analyses including counting puncta, measuring area of individual puncta of neuronal cell bodies, and densitometric analyses of neuritic α-syn clearly revealed that de novo expression of bpoz-2 indeed ameliorated the burden of α-syn in both SNpc and SNpr regions. On the other hand, lentiviral administration of bpoz-2 shRNA upregulated the expression of α-syn in the cell bodies and fibers of nigral DA neurons of A53T-tg animals, which we confirmed by immunohistochemical and immunoblot analyses. Moreover, shRNA-mediated knocking down of bpoz-2 significantly attenuated the dimeric, trimeric, and oligomeric inclusions of α-syn suggesting that BPOZ-2 is directly involved in the amelioration of α-syn suggesting the absence of bpoz-2 is crucial for the development of α-syn pathology. Degradation of protein occurs by two different metabolic pathways, either via ubiquitin–proteasome pathway (UPS) or autophagy-lysosome pathway. Several studies have attempted to investigate a link between α-syn and the proteasome^24^,^25^; however findings show that mutant α-syn can reduce the net proteasomal activity in living cells^26^. Importantly, recent studies suggest that soluble oligomeric intermediates of α-syn might specifically impair the function of the 26S proteasome indicating that ubiquitin–proteasome pathway might not be involved in the degradation of α-syn. Parkin is the only established E3-ubiquitin ligase in PARK family of proteins. Therefore, next we tested if BPOZ-2 was involved in an interaction with parkin in order to ameliorate α-syn. However, according to our IP analysis, we were unable to observe any interaction between parkin and BPOZ-2 nullifying the involvement of E3–ubiquitin pathway for the clearance of α-syn in nigral DA neurons. In order to establish the interaction between BPOZ-2 and other PD–related proteins, we used functional protein association network STRING, a huge database that predicts protein-protein interaction based on their cross-talk with other signaling molecules. Interestingly, our search resulted that BPOZ-1 (or ABTB1), a splice variant of BPOZ-2, could be involved in protein-protein interaction with two PD-related proteins PINK1 and DJ-1 (PARK7. Since, activation of PINK1 had been shown to ameliorate α-syn aggregation^27^,^28^, our STRING analysis intrigued us to find out if there was any interaction existed between PINK1 and BPOZ-2. On the other hand, similar IP analysis revealed that there was an interaction between BPOZ-2 and PINK1 suggesting that mitochondrial autophagic pathway might be involved in the clearance of α-syn. Moreover, we observed that the interaction between PINK1 and α-syn had been strongly induced in the nigra of lenti-*bpoz-2 animals, whereas the interaction between these two protein had been markedly reduced in the nigra of lenti-shbpoz-2 animals suggesting that bpoz-2 plays a crucial role in the interaction between PINK1 and α-syn in the nigra that might lead to the autophagic degradation of α-syn.

Overall, our present manuscript highlighted the novel role of an ankyrin-rich and BTB domain containing protein BPOZ-2 in the amelioration of α-syn. However, whether knocking down of bpoz-2 is sufficient to establish α-syn pathology remains to be elucidated. Therefore our future aim is to understand if there is a spontaneous accumulation of α-syn in the nigra of bpoz-2 knock-out mice. Moreover, its role in PINK1-mediated autophagic clearance of αsyn is not clear yet. Although, we have observed that there is interaction between these BPOZ-2 and PINK1 and the interaction between α-syn and PINK1 has been significantly enhanced with the *de novo* overexpression of bpoz-2, the direct role of BPOZ-2 in the autophagic clearance of α-syn needs to be evaluated. Upregulation of autophagic markers and enhanced autophagic activity in the nigra of lenti-*bpoz-2 animals would definitely prove that ectopic overexpression of BPOZ-2 definitely plays role in the autophagic clearance of α-syn. Similarly, the downregulation of autophagic markers and decreased autophagic activity in lenti-shbpoz-2 animals would indicate that silencing of endogenous bpoz-2 gene would be crucial for the reduced autophagic clearance of α-syn. We would also test if BPOZ-2 gene delivery could improve the amelioration of other Lewybody (LB) components including Amyloid and Tau aggregates in AD brain.

## Methods

### Reagents and Animals

Anti-rabbit Bpoz-2 antibody (WB, IHC and IP; cat # ab112610), anti-rabbit αsyn antibody (cat # ab32518) and anti-PINK1 antibody (WB and IP: cat # ab75487) were purchased from Abcam. Anti-sheep α-syn antibody (Cat# AB5336P) and Rabbit-anti Tyrosine Hydroxylase (TH) antibody (Cat # AB152) were purchased from EMD Millipore. Bpoz-2 overexpression construct (cat # EX-Mm12657-Lv104), Bpoz-2-siRNA (cat# MSH042485-LvH1), lentiviral packaging kit, HEK-293Ta cells (Cat # CLv-PK-01), and chemically competent E-Coli cells (Cat # STK200-10) were purchased from Genecopoeia. C1A53T-tg animals (strain # B6;C3-Tg(Prnp-SNCA*A53T)83Vle/J) were purchased from Jackson Laboratory. These mice were genotyped and maintained in the animal care facility of Rush University Medical Center up to 8–9 moths of their age before viral injection. Animal experiments and viral injections had been performed in accordance with the guidelines and regulations imposed by Institutional Animal Care and Use Committee (IACUC) and Institutional Biosafety Committee (IBC) respectively. Experiments were performed after receiving necessary approvals from IACUC (protocol # 14-028) and IBC (protocol # 040814).

### Lentiviral packaging of plasmids and injection to the nigra

Lentiviral plasmids were enriched by E-coli transformation, packaged in HEK-293Ta cells using lentiviral packaging mix as described in manufacturer’s protocol^20^. Eight to nine months old male A53T-tg mice were injected with lentiviral particles (10^7^/uL) of bpoz-2 overexpression and bpoz-2-shRNA constructs in their nigra. Briefly, an injection cannula (26.5 gauge) was applied stereotaxically into the substantia nigra (anteroposterior, −3.0 mm from bregma; mediolateral, 1.2 mm; dorsoventral, 4.3 mm). The infusion was performed at a rate of 0.2 ml/min and wound healing and recovery was monitored after the injection was done.

### Immunohistochemical analyses

two months after injection, mice were sacrificed and their brains fixed, embedded, and processed for tyrosine hydroxylase (TH) (antibody dilution: 1:500) and α-syn (antibody dilution: 1:500) immuno-staining using a protocol described elsewhere^29^.

### Immunoblot and densitometric analyses

Immunoblot analysis for α-syn was carried out as described earlier^29^. Briefly, cell homogenates were electrophoresed, proteins were transferred onto a nitrocellulose membrane, and bands were visualized with an Odyssey infrared scanner after immunolabeling with respective primary antibodies followed by infra-red fluorophore-tagged secondary antibody (Licor-Odyssey). Densitometric analyses were performed in image J software and plotted after normalizing with actin.

### HPLC Analysis

Striatal tissues were sonicated in 0.2 M perchloric acid containing isoproterenol (internal standard) and resulting homogenates were centrifuged at 20,000 g for 15 min at 4 °C. After pH adjusting and filtration, 10 μL of supernatant was injected onto a Eicompak SC-3ODS column (HPLC-ECD System EiCOMHTEC-500 from JM Science) and analyzed as described elsewhere^29^.

### Behavioral Analysis

Animals used in this study are 8–9 months old male A53T (strain # B6;C3-Tg(Prnp-SNCA*A53T)83Vle/J) receiving different lentiviral constructs of bpoz-2 (n = 3 per group). These animals were housed in ventilated micro-isolator cages in an environmentally controlled vivarium (7:00 A.M. /7:00P.M. light cycle; temperature maintained at 21–23 °C; humidity 35–55%). Animals are provided standard mouse chow and water ad libitum and closely monitored for health and overall well-being daily by veterinary staff and the investigator. Two months after viral manipulation, animals were placed individually into an open field arena made of plexiglass cage [42 cm (L) × 42 cm (W) × 32 cm (H)]. In this procedure, the open field environment is much larger than that of the home cage, and is unfamiliar to the animal. Therefore, in the beginning of experiment animals were allowed to adapt with the environment for 10 mins. After that, animal’s open field behavior was recorded. Each animal was monitored for three times with 5 minutes acquisition and 5 minutes of interval time. Motor activity is monitored in the Digiscan Open Field Activity System (Omnitech Electronics). In this system, the plexiglass cage is surrounded by a frame consisting of numerous photocells that continuously tracks the animal’s movement. Data are collected in the form of photobeam breaks as an indication of activity using Digiscan analyzer recording unit (Omnitech Electronics). During interval time, digital recording unit had been switched off; however animals were allowed to stay inside the plexiglass cage for the entire test. Test cage was cleaned with alcohol after replacing each animal. In the open-field experiment, various parameters, such as horizontal activity, total distance, number of movements, movement time, rest time, stereotypy counts, rearing, mean distance, meantime, center distance, and center time, were measured accordingly. In rotarod, animals were allowed to run on a motorized backward moving platform (Med associates Inc) at a constant speed of 24 rpm with cut-off time 5 minutes. To eliminate stress and fatigues, mice were given a 5-min rest interval. Each animal was recorded three times.

### Software and statistical analyses

Counting of α-syn puncta was performed after reconstructing individual α-syn-ir DA cell body in surfer 12 contouring software manufactured by Golden Software, Colorado followed by counting individual puncta in touch counting module of Olympus Microsuite software. Co-localization of TH and BPOZ-2 in nigra was performed using NIH FIJI Co-localization tool. Quantitative data was presented as the mean ± SEM. Statistical significance was accessed via an unpaired two-tailed Student’s t test or an ANOVA test with Student-Newman-Keuls post hoc analysis.

## Additional Information

**How to cite this article**: Roy, A. *et al.* BPOZ-2 Gene Delivery Ameliorates Alpha-Synucleinopathy in A53T Transgenic Mouse Model of Parkinson’s Disease. *Sci. Rep.* **6**, 22067; doi: 10.1038/srep22067 (2016).

## Supplementary Material

Supplementary Information
