# Supplementary material for: BPOZ-2 Gene Delivery Ameliorates Alpha-Synucleinopathy in A53T Transgenic Mouse Model of Parkinson’s Disease
Source: Sci Rep. 2016 Feb 26;6:22067. doi: 10.1038/srep22067 (PMC4768134; doi:10.1038/srep22067)
Supplement: Supplementary Information [file srep22067-s1.pdf]

BPOZ-2 Gene Delivery Ameliorates Alpha-Synucleinopathy in A53T Transgenic Mouse Model of Parkinson's Disease

***Avik Roy*[1], *Suresh Babu Rangasamy*[1], *Madhuchhanda Kundu*[1], *Kalipada Pahan*[1, 2]**

[1]Department of Neurological Sciences, Rush University Medical Center, Chicago, IL; [2]Division of Research and Development, Jesse Brown Veterans Affairs Medical Center, 820 South Darnen Avenue, Chicago, IL

Running title: *BPOZ-2 ameliorates alpha-synuclein in nigra*

To whom correspondence should be addressed:

Avik Roy, Ph.D.
Department of Neurological Sciences
Rush University Medical Center
1735 West Harrison St, Suite 320
Chicago, IL 60612
Telephone (312) 942-8274
Fax (312) 563-3571
Email: avik_roy@rush.edu

Supplementary Information

Supplementary Figure 1. HPLC-based dopamine Analysis in the striatum and behavioral analyses of A53T-tg animals receiving vector, lenti-*bpoz-2 and lenti-shbpoz-2 genes.(A) Horizontal activity, (B)total distance, (C)stereotypy counts, and (D) rotarod activity were measured in two different groups. Results were analyzed as mean ± SEM of three different animals per group. Statistical difference was measured by one way-ANOVA considering the genetic manipulation as an effector ($p<0.05$). No significance was found among different experimental groups. (E) Chromatograms for the detection of dopamine (enclosed in the dotted box) in vector (top), lenti-*bpoz-2 (middle) and lenti-shbpoz-2 (bottom) striatal homogenates. Y axis = voltage unit; X axis = time of detection. (F) Quantification of dopamine in the striatal homogenates of A53T animals with different bpoz-2 gene constructs. Results are mean ± SEM of three different animals.

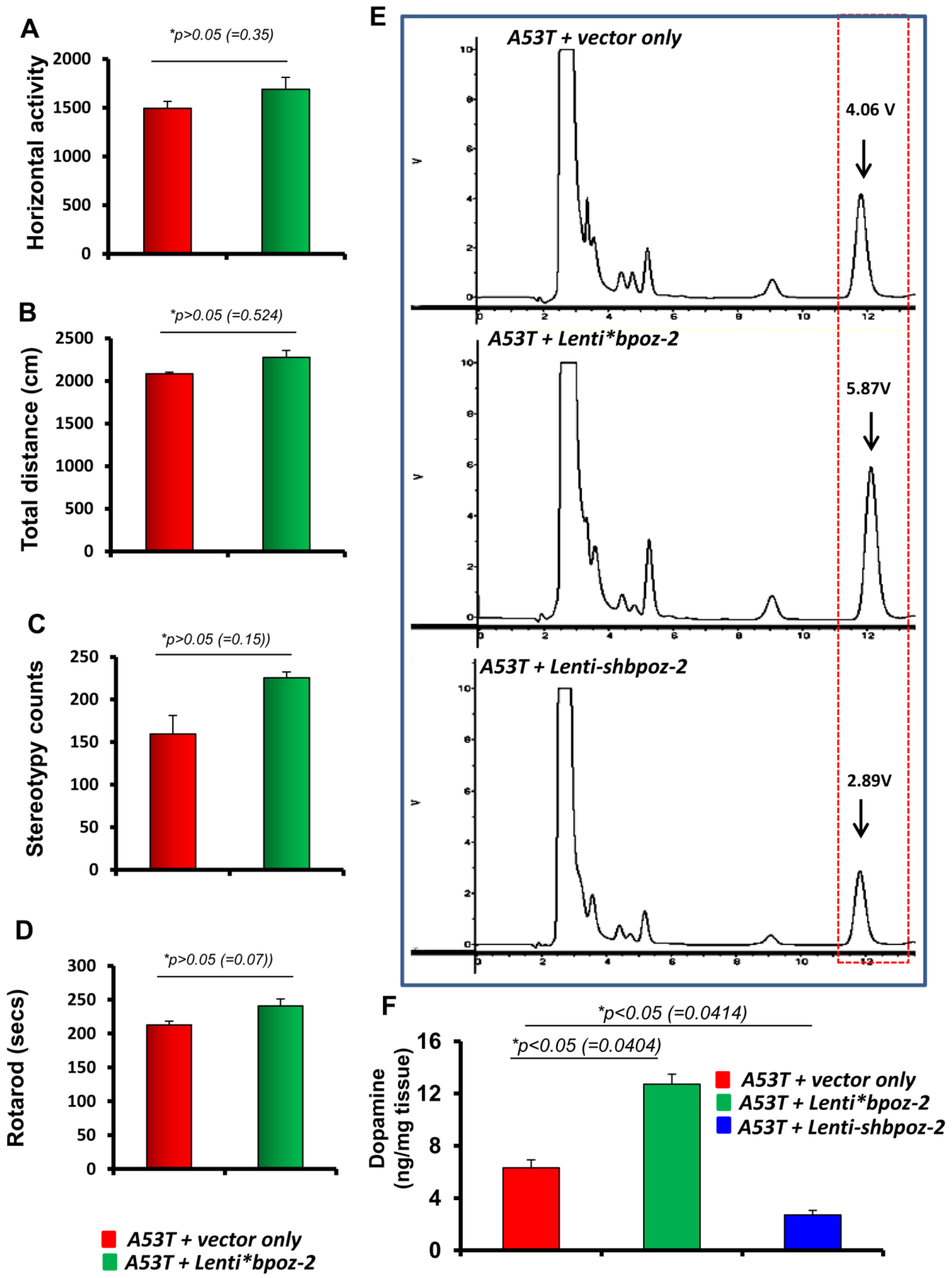
A
*p>0.05 (=0.35)
Horizontal activity
2000
1500
1000
500
0
B
*p>0.05 (=0.524)
Total distance (cm)
2500
2000
1500
1000
500
0
C
*p>0.05 (=0.15))
Stereotypy counts
250
200
150
100
50
0
D
*p>0.05 (=0.07))
Rotarod (secs)
300
250
200
150
100
50
0
A53T + vector only
A53T + Lenti*bpoz-2
E
A53T + vector only
4.06 V
A53T + Lenti*bpoz-2
5.87V
A53T + Lenti-shbpoz-2
2.89V
F
*p<0.05 (=0.0414)
*p<0.05 (=0.0404)
Dopamine (ng/mg tissue)
16
12
8
4
0
A53T + vector only
A53T + Lenti*bpoz-2
A53T + Lenti-shbpoz-2
